# Combining multi-objective genetic algorithm and neural network dynamically for the complex optimization problems in physics

**DOI:** 10.1038/s41598-023-27478-7

**Published:** 2023-01-17

**Authors:** Peilin Wang, Kuangkuang Ye, Xuerui Hao, Jike Wang

**Affiliations:** The Institute for Advanced Studies, Wuhan University, Wuhan, 430072 China

**Keywords:** Techniques and instrumentation, Applied physics

## Abstract

Neural network (NN) has been tentatively combined into multi-objective genetic algorithms (MOGAs) to solve the optimization problems in physics. However, the computationally complex physical evaluations and limited computing resources always cause the unsatisfied size of training set, which further results in the combined algorithms handling strict constraints ineffectively. Here, the dynamically used NN-based MOGA (DNMOGA) is proposed for the first time, which includes dynamically redistributing the number of evaluated individuals to different operators and some other improvements. Radio frequency cavity is designed by this algorithm as an example, in which four objectives and an equality constraint (a sort of strict constraint) are considered simultaneously. Comparing with the baseline algorithms, both the number and competitiveness of the final feasible individuals of DNMOGA are considerably improved. In general, DNMOGA is instructive for dealing with the complex situations of strict constraints and preference in multi-objective optimization problems in physics.

## Introduction

Multi-objective genetic algorithms (MOGAs) such as NSGA-II^1^, MOEA/D^2^, SPEA^3^ have shown good performance in many engineering optimization problems. Being inspired by the evolutionary theory of “survival of the fittest”, the competitive individuals can be obtained through operators of selection, mutation, and crossover by iteration. These individuals that cannot outperform each other on all objectives create a set, the so-called nondominated front. In terms of the physical optimization problems in which the evaluations are always computationally complex, their population sizes in MOGAs are generally small because of the limited computing resources. When the parameter space formed by the decision variables is very large and local optima exist in these questions, these algorithms tend to converge on local optima rather than global optima.

The field of accelerator can be taken as an example. There are many complex optimization problems that do not have the known optimal solution sets (the Pareto front) in accelerator fields, and good performance has been preliminarily obtained with MOGAs in some of these problems, such as the optimization of lattice^4^, Free Electron Laser^5,6^ and other accelerator facilities^7^. Designing the shape of radio frequency (RF) cavity is a sort of important problem as well, in which several objectives such as geometric shunt impedance (R/Q), Q factor and shunt impedance (R_a_) are optimized simultaneously by tuning the geometric parameters of the cavity. At the same time, an equality constraint^8^ has to be considered in this design, which means the frequency of fundamental mode (*f*_FM_) of cavity must be equal to a given target frequency, otherwise the cavity cannot be used even with great performance. These individuals that satisfy constraint are called feasible individuals, while others are infeasible. Although MOGAs have obtained some competitive individuals in RF cavity design^9–11^, these works still rely on the professional knowledge of manual process to set the small and proper decision space. As a result, the manual optimization process as the most popular method in engineering^12,13^ cannot be replaced by MOGAs yet.

Neural network (NN) has been tentatively combined into MOGAs to increase the population size and speed up convergence^14–16^. In these attempts, the common idea (named NBMOGA) is estimating all the individuals with NN instead of evaluating them directly, while the difference lies in the composition of training set and the number of times the NN is trained. In a specific algorithm^15^, after executing standard NSGA-II for several generations, NN is trained to estimate more individuals of which some are selected to be further evaluated. This combination performs good in the optimization of the dynamic aperture area and the Touschek lifetime. When the former objective remains unchanged, the latter one increases about 10% compared with the standard MOGA in similar time. It is clearly seen that NBMOGA do well in the convergence speed, but the shortcoming of entirely relying on the estimated indicators to select parents since the training of NN begins^14–16^ still exists in them. Once the strict constraint is considered in the optimization, the feasible individuals are easily estimated as infeasible because of the small size of training set, and this is a major challenge for these algorithms.

To deal with this problem, a penalty operation that can be progressively stricter over generations is executed in fitness function to fulfill constraints gradually. Meanwhile, NN is included in an operator. This operator not only produces several individuals to be further evaluated, just like the operators of mutation and crossover do, but also screens in a great number of estimated individuals internally. The performance of these operators is different when the penalty changes, so the number of individuals that come from these operators to be further evaluated is dynamically redistributed. Namely, this algorithm is called the dynamically used NN-based MOGA (DNMOGA). In addition, accessibility algorithm is proposed as a new idea to deal with preference in NSGA-II, which doesn’t depend on extra reference points that are set manually in other algorithms^17,18^ to lead the nondominated front to approach them.

The shape of spherically shaped (SS) normal conducting cavity, a kind of RF cavity used in PEP II^19^, is optimized to prove the advantage of DNMOGA. Differences among various algorithms are compared, while two NN models are combined into DNMOGA respectively to illustrate the relationship of the performance of this optimizer and the accuracy of NN. Besides, some other details about DNMOGA are also discussed below. As a result, DNMOGA shows the potential to completely replace manual procession in this question, which also announces its ability to solve other optimization problems in physics that have the similar features with this design.

## Results

### NN models

**Figure 1:**
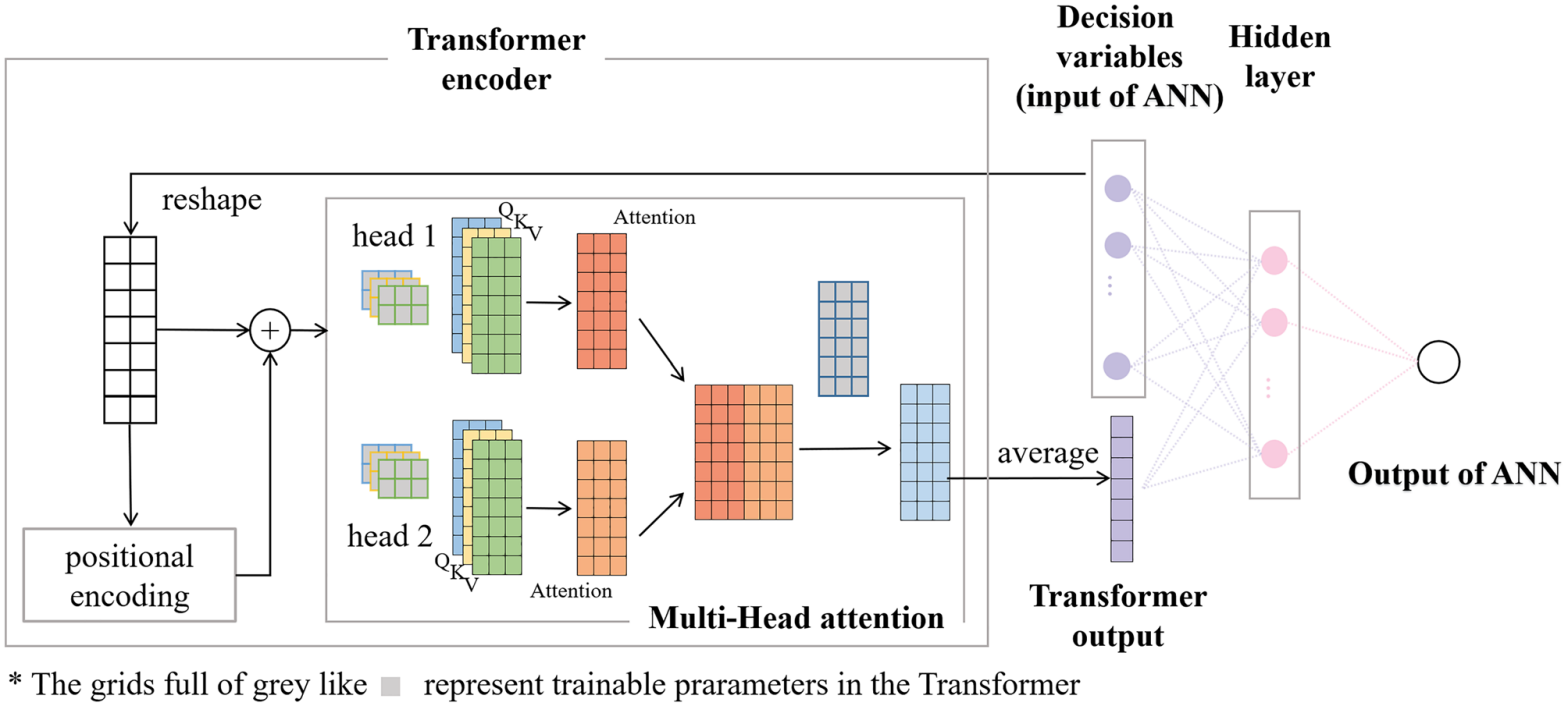
The model of NN combining ANN and Transformer.

One of the two NN models mentioned above is a simple artificial neural network (ANN) that has 5 neurons within one hidden layer^20–23^. The other one, which is shown in Fig. 1, combines ANN and Transformer^24,25^ and has better accuracy. Note that neither of these models can accurately estimate the indicator of equality constraint in these experiments. The accuracies of these models are expressed with R^2^, a criterion unrelated to MOGAs. This criterion is defined as

1 $$R^{2} = { }1 - \frac{{\sum \left( {y_{i} - \hat{y}_{i} } \right)^{2} }}{{\sum \left( {y_{i} - \overline{y}} \right)^{2} }} ,$$

where ${y}_{i}$ is the label, ${\widehat{y}}_{i}$ is the estimated value and $\overline{y }$ is the average of ${y}_{i}$. The indicator estimated by NN is more precise when the value of R^2^ is closer to 1. The values of R^2^ of these models in 5 indicators are listed in Table 1. The 5 indicators are *f*_FM_, R/Q of the FM (R/Q_FM_), R_a_ of the FM (R_a FM_), Q factor of the FM (Q_FM_), frequency of the higher order modes (*f*_HOM_), in turn. From this table, the Transformer combined with ANN estimates more accurately in all indicators. The specific illustration of these models and the idea of designing these models are described in Supplementary materials.

**Table 1:** The values of R^2^ in different NN models and indicators are compared.

Training sets and model		Number of trainable parameters	R^2^				
			${f}_{\mathrm{FM}}$	${R/Q}_{\mathrm{FM}}$	${R}_{\mathrm{a FM}}$	${\mathrm{Q}}_{\mathrm{FM}}$	${f}_{\mathrm{HOM}}$
3000 samples	Transformer with ANN	265	0.987	0.985	0.989	0.988	0.984
	2-layers ANN	76	0.923	0.954	0.957	0.969	0.942

### Fitness function for dealing with constraint

Although feasible individuals are the favorites to decider, infeasible individuals can always provide the hidden information about global optima and the lower constraint violation (cf. Eq. 9). So, how to balance the numbers of feasible and infeasible individuals in parents is an important problem. Generally, there are four categories^26^ to deal with it: adding a penalty to fitness function^27,28^, judging constraint condition before adding the penalty^29,30^, proposing novel selection strategy^31–33^ and regarding constraint as objective^34,35^. The popular ways in engineering are the first two categories, while the specific operation of penalty in them is punishing infeasible individuals by increasing their fitness. Unfortunately, the uncontrollable numbers of feasible and infeasible parents in these algorithms can be dramatically different when the range of constraint changes or the number of generations is different, which results in a failure of balance.

To solve the mentioned problem, the feasible and infeasible parents are put into two independent groups as a detail of setting parents in this work, while a fitness function with a penalty operation that can make individuals fulfill constraints gradually is used. In the mathematical model shown below, $\overrightarrow{x}={\left({x}_{1},{x}_{2},\dots ,{x}_{l}\right)}^{ }\in S$ is a *l*-dimensional decision vector, in which $S\subset {\mathbb{R}}^{l}$ is the decision space. $\overrightarrow{F}\left(\overrightarrow{x}\right)$ is a fitness vector consisting of the fitness values of *m* objectives, and ${F}_{i}\left(\overrightarrow{x}\right)(i\in \{\mathrm{1,2},\dots ,m\})$ is the fitness function. The ${g}_{j}\left(\overrightarrow{x}\right)$ and ${h}_{j}\left(\overrightarrow{x}\right)$ are inequality and equality constraints respectively, *n* represents the total number of inequality and equality constraints, and ${x}_{q}^{min}$ and ${x}_{q}^{max}$ are the bound constraints of ${x}_{q}^{ } (q\in \{\mathrm{1,2},\dots ,l\})$.

2 $$\begin{gathered} \begin{array}{*{20}c} {\min } & {\vec{F}\left( {\vec{x}} \right) = \left( {F_{1} \left( {\vec{x}} \right),F_{2} \left( {\vec{x}} \right), \ldots ,F_{m} \left( {\vec{x}} \right)} \right)^{T} } \\ \end{array} { } \hfill \\ {\text{ s}}.{\text{t}}.{ }\left\{ {\begin{array}{*{20}c} { g_{j} \left( {\vec{x}} \right) \le 0, j = 1, \ldots ,p } \\ {{ }h_{j} \left( {\vec{x}} \right) = 0, j = p + 1, \ldots ,n} \\ {x_{q}^{min} \le x_{q}^{ } \le x_{q}^{max} ,q = 1,2, \ldots ,l } \\ \end{array} } \right.{ }. \hfill \\ \end{gathered}$$

The general way to penalize the fitness function is shown here^27,30^:

3 $${ }F_{i} \left( {\vec{x}} \right){ } = 1 - f_{i} \left( {\vec{x}} \right) + \mathop \sum \limits_{j = 1}^{n} r_{j}^{^{\prime}} c_{j} \left( {\vec{x}} \right)$$

4 $$c_{j} \left( {\vec{x}} \right) = \left\{ {\begin{array}{*{20}c} {\max \left( {0,{ }g_{j} \left( {\vec{x}} \right)} \right),{ } j = 1, \ldots ,p } \\ {\max \left( {0,\left| {h_{j} \left( {\vec{x}} \right)} \right| - \delta } \right),j = p + 1, \ldots ,n} \\ \end{array} } \right.,$$

where ${f}_{i}\left(\overrightarrow{x}\right)$ is the value of the *i*th objective, and $\delta$ is a very small positive tolerance value to relax the equality constraints. ${r}_{j}^{^{\prime}}$ as the penalty coefficient used to be adjusted in different problems to keep the balance^28,36^. Based on these experiences, the ${r}_{j}^{^{\prime}}$ is redesigned to match the operator with NN, and the whole expression of penalty operation is described below.

The ${F}_{i}\left(\overrightarrow{x}\right)$ used in Eq. (2) is set as

5 $${ }F_{i} \left( {\vec{x}} \right){ } = 1 - e_{i} \left( {\vec{x}} \right) + d\left( {\vec{x}} \right){ }$$

6 $$e_{i} \left( {\vec{x}} \right) = \left\{ {{ }\begin{array}{*{20}c} {\begin{array}{*{20}c} {f_{i} \left( {\vec{x}} \right) \cdot r_{j} ,} & {CV\left( {\vec{x}} \right) > 0} \\ \end{array} } \\ {\begin{array}{*{20}c} {{ }f_{i} \left( {\vec{x}} \right),} & {{ }CV\left( {\vec{x}} \right) = 0} \\ \end{array} } \\ \end{array} } \right.$$

7 $$d\left( {\vec{x}} \right) = \left\{ {\begin{array}{*{20}c} { \begin{array}{*{20}c} {CV\left( {\vec{x}} \right) \cdot \left( {1 - r_{j} } \right),} & {CV\left( {\vec{x}} \right) > 0} \\ \end{array} } \\ {\begin{array}{*{20}c} {{ }0,{ }} & {{ }CV\left( {\vec{x}} \right) = 0} \\ \end{array} } \\ \end{array} } \right.$$

where $\mathrm{CV}\left(\overrightarrow{x}\right)$ described in Eq. (9) is the overall value of constraint violation, and ${r}_{j}$ is a penalty coefficient shown in Eq. (8).

8 $${{r}_{j} =\left(\frac{1}{1+{k}_{j}\cdot const\frac{pre}{total}}\right)}^{4}$$

In Eq. (8), $pre$ represents the number of generations (the evaluated generations, Fig. 3) at present, and $total$ represents the total number of evaluated generations. ${k}_{j}$ is an adjustable value and written as *k* later, because there is only one constraint in the specific problem. $const$ is a constant to keep a suitable range of speed to converge, and it is set as $\frac{1}{{e}^{4}}$. In this penalty operation, ${F}_{i}\left(\overrightarrow{x}\right)$ will be more and more large when $\mathrm{CV}\left(\overrightarrow{x}\right)$ grows. ${r}_{j}(pre)$ has a good mathematical property, because it is a continuous and derivable convex function when $pre$ is regarded as an independent variable.

The $\mathrm{CV}\left(\overrightarrow{x}\right)$ is calculated as

9 $$\mathrm{CV}\left(\overrightarrow{x}\right)=\sum_{j=1}^{n}{r}_{j}\cdot {c}_{j}\left(\overrightarrow{x}\right)$$

where the operation of normalization can be calculated according to Eq. 10.

Note that the values of ${F}_{i}\left(\overrightarrow{x}\right)$, $CV\left(\overrightarrow{x}\right)$ and ${f}_{i}\left(\overrightarrow{x}\right)$ must be normalized. In each generation, the values that come from the same function create a set, and the method of normalization in each set is expressed below:

10 $$\mathrm{norm}\left(Y\right)= \frac{Y-\mathrm{min}(Y)}{\mathrm{max}\left(Y\right)-\mathrm{min}(Y)} ,$$

where the $Y$ is an element in set, and $\mathrm{max}\left(Y\right)$, $\mathrm{min}(Y)$ is the maximum and minimum in this set respectively.

### Dynamically redistributing the numbers of individuals to operators

The way to redistribute is defined as

11 $${{N}_{v+1}}_{ }=T\times \frac{{{PN}_{ }}_{v}}{{{PT}_{v}}_{ }} .$$

In Eq. 11, $T$ is fixed as the total number of evaluated individuals in each generation, while ${{PT}_{v}}$ represents how many individuals are selected as parents totally in the *v*th generation. For each operator, ${{N}_{v+1}}$ represents the redistributed number of individuals in the (*v* + 1) the generation, and ${{{PN}_{ }}_{v}}$ represents how many individuals from this operator are selected as parents in the *v*th generation.

### The accessibility algorithm

Sometimes the decider might rank the degrees of importance to all objectives, which means preference exists. The common idea to deal with preference is introducing manual reference points^17,18,37^ and calculating distances between individuals and these points, then leading the nondominated front to approach them. But these methods only concentrate on the narrow regions around the reference points where too much hidden information about global optima is lost, and consequently converge to local optima in complex problems. In DNMOGA, accessibility which is frequently used in traffic problems^38^ is introduced instead of original crowding-distance, and it can be expressed as

12 $${}_{1}{}^{ }A=\sum_{o=1}^{M}{}_{1}{}^{ }{A}_{o}=\sum_{o=1}^{M}\frac{{S}_{o}}{{T}_{1,o}^{Y}}=\sum_{o=1}^{M}\frac{\overrightarrow{H}\cdot {{\overrightarrow{F}}_{o}}^{ }+1}{{D}_{1,o}} .$$

The first three terms of Eq. 12 is expressed in^38^ as well, in which the $M$ represents the number of areas, ${}_{1}{}^{ }A$ is the accessibility of the first area, and ${}_{1}{}^{ }{A}_{o}$ is the accessibility from the first area to the *o*th. At the same time, ${S}_{o}$ represents the vitality in the *o*th area, and it can be described as population, gross domestic product, etc. Besides, ${T}_{1,o}$ represents the travel time between these areas, while Y is an exponent to describe the effect of travel time. In DNMOGA, ${T}_{1,o}^{Y}$ is replaced by ${D}_{1,o}$ to represent the Euclidean distance of objectives between individuals. Then, accessibility can be used to deal with preference by relating ${S}_{o}$ to every objective as a number larger than 1. In the last term of Eq. 12, $\overrightarrow{H}$ represents the preference vector with a series of values, and each of them is corresponded to a specific objective. The larger value in $\overrightarrow{H}$ represents that the corresponding objective is more important. In addition, the ${\overrightarrow{F}}_{o}$ represents the fitness vector for these objectives.

**Figure 2:**
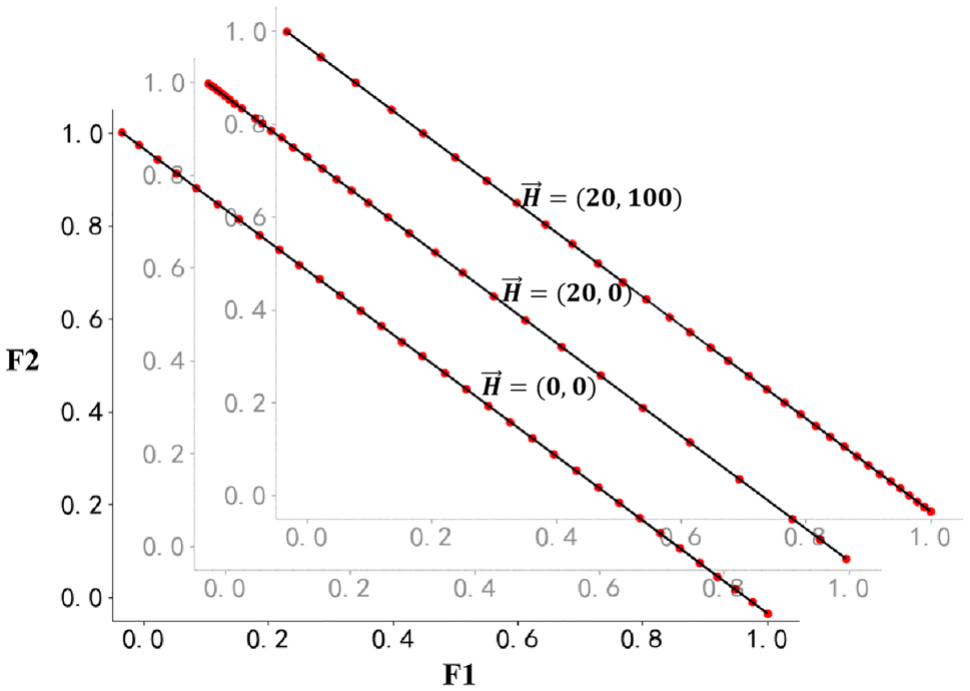
The distribution of selected individuals which is obtained by using accessibility algorithm. 1000 nondominated individuals are set and 33 individuals are selected. There are two objectives: F1 and F2, and both should be minimized. When the $\overrightarrow{H}$ is set as (0, 0), it means no preference in F1 and F2, and the distribution of selected individuals is very uniform. When the $\overrightarrow{H}$ is (20, 0), the individual with lower F1 score prefers to be taken. When the $\overrightarrow{H}$ is (20, 100), although preference value of F1 is 20, but F2 has higher preference value, and more individuals good at F2 are taken.

There are two sets in accessibility algorithm, one is the pending set in which all the individuals of nondominated front are initially included as the pending parents of the next generation, and the other one is the selected set to collect the selected individuals. After normalizing the objective value and putting the boundary solutions to the selected set from the pending one, the accessibility of each pending individual to all the selected individuals are calculated, then the most inaccessible one is taken to the selected set. This procession is repeated until the size of selected set is satisfied. The picture below (Fig. 2) shows the advantage of accessibility. The fake code is released in Supplementary materials.

### The complete process of DNMOGA

**Figure 3:**
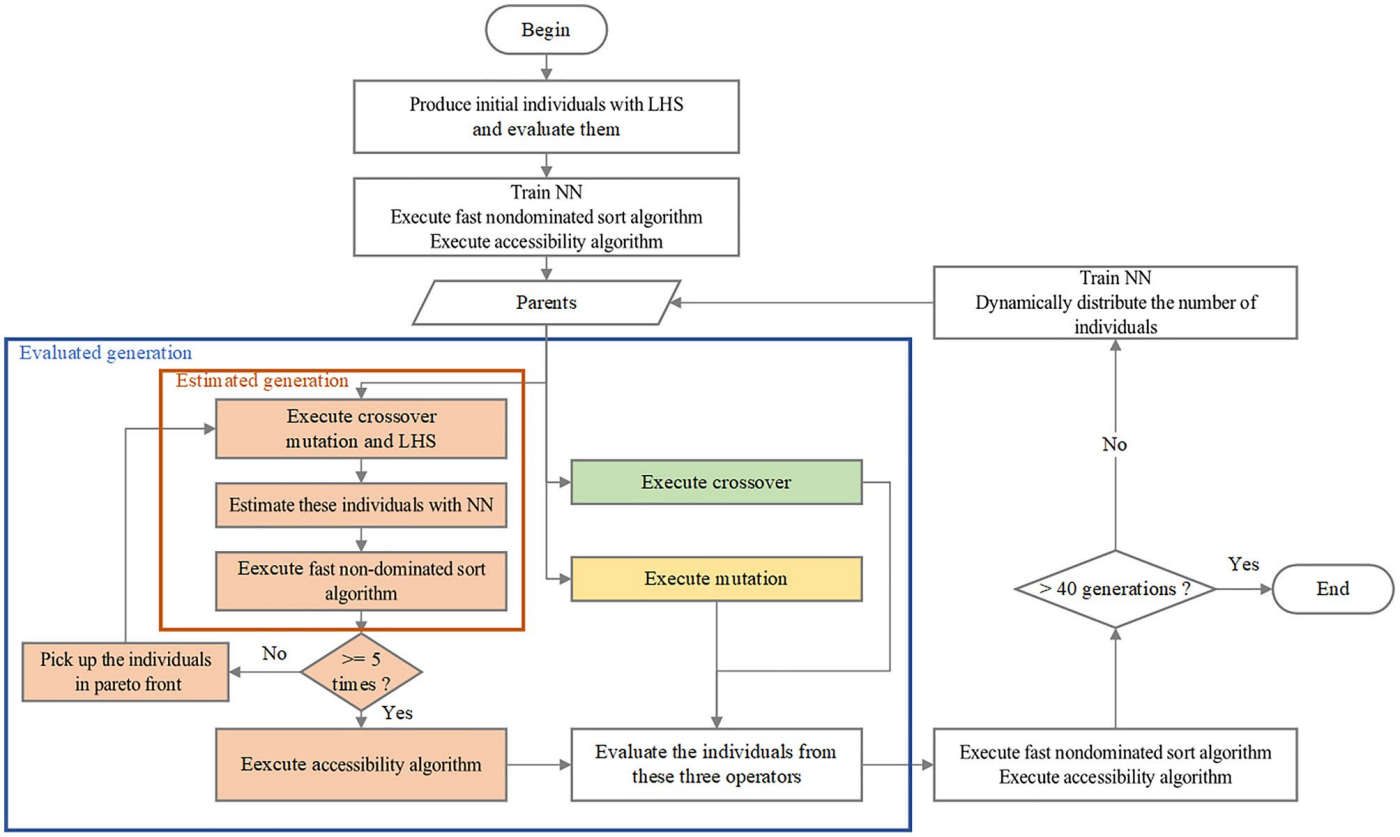
The flow chart of DNMOGA. The green, yellow, and orange blocks represent the three operators, and they are named as crossover, mutation, and the operator with NN, respectively.

The flow chart of DNMOGA is shown in Fig. 3. Before describing the complete process of DNMOGA, it is necessary to detail the operator including NN first. In this operator (the orange blocks in Fig. 3), the crossover, mutation and Latin hypercube sampling (LHS)^39^ are used to produce astronomical individuals together, then NN is used to estimate the indicators of these individuals. After executing the fast nondominated sort algorithm, the nondominated front is generated as the parents of next generation. This series of operations constitutes the estimated generation, which is carried out five times continuously. Then the accessibility algorithm is executed among the nondominated front of the fifth estimated generation to pick up a certain number of individuals that will be further evaluated.

The first step of the complete process in DNMOGA is evaluating a certain number of individuals that are produced by LHS as the training set of NN and the first generation of NSGA-II, after which the parents are selected and the main loop begins. In this cycle, crossover and mutation are used again as two of the operators (the green and yellow blocks in Fig. 3), and they produce the individuals to be directly put in the actual evaluator together with the operator with NN. Then, all the evaluated individuals in this generation are mixed with these earlier evaluated, after that the fitness function is executed. Once the fast nondominated sort algorithm and accessibility algorithm are executed in feasible and infeasible individuals respectively, the two groups of parents for next generation can be obtained. As the last step, the numbers of individuals generated by these operators in the next generation are dynamically redistributed. This generation produces new individuals through actual evaluator, and it is naturally called the evaluated generation. The fake code of the complete process is released in Supplementary materials.

In this study, some specific details can also be helpful to obtain the competitive individuals. To begin with, as an assisting method to satisfy constraint, standard mutation operation is replaced by adjusting the *f*_FM_ to produce more feasible individuals, and the specific operation of adjusting frequency is shown below:

13 $$Req\mathrm{^{\prime}}= \frac{{f}_{FM}-499.65}{2.55}+Req .$$

It is assumed that *f*_FM_ is only related to $Req$, and because of the linear relationship in physics between Req and *f*_FM_, an appropriate coefficient is empirically set as 2.55 [MHz/mm]. The $Req\mathrm{^{\prime}}$ in Eq. 13 is the new decision variables obtained by this operation. The comparison between this method and the standard mutation will be described blow. For the optimization problems in physics, tuning frequency might not suit for all scenarios, but the relationship between the objectives and decision variables can be expressed in a formula, and the positive or negative correlation can be found locally as the basis to design the assisting method even if it sometimes is hard for the objectives to be calculated correctly according this relationship. The second detail is about the two groups of parents. To increase the variety of parents, the number of parents in each group is the greater value between 90% of the size of their nondominated front and a constant. Note that all the values mentioned below about the parents are this constant, and the operation of two groups of parents is not used in selecting estimated individuals because of the dissatisfied accuracy.

### Optimization of the SS cavity

The shape and the geometric parameters of the SS cavity are shown in Fig. 5c, in which the tube parameters *Rt* and *Lt* that we do not care about are fixed as 200 and 16 mm respectively.

In order to obtain the shapes with performance as good as possible, larger space should be explored, which means the limits among the geometric parameters must be considered. For example, the *R0_l*, *R0_r* should be smaller than Req, while the sum of *Rt*, *R3_l* and nose should be smaller than the *Req* as well. The way to deal with these limits is executing nonlinear transformations to relate the geometric parameters with independent variables, and the variables should have the same degrees of freedom to the parameters. As a result, 13 independent variables are set as decision variables in DNMOGA. The specific procession is discussed in Supplementary materials, and the range of *Leq* is in the range of 11 and 630 mm, while *Req* is between 116 and 216 mm.

Besides the indicators mentioned in the introduction, there are also many other indicators in the optimization problems of RF cavity, such as the Q factor, the normalized peak electric field on the cavity surface, and so on. Principally, Q factor and R_a_ of the FM should be maximized, but according to the previous papers^9,10,40^ and mathematical relationship among ${R/Q}$, ${R}_{\mathrm{a}}$ and Q factor (as shown in Eq. 14), R_a_ and ${R/Q}$ are usually considered as the objectives. In addition, the normalized peak electric field on the surface of the optimized normal conducting cavity can always satisfy the decider’s requirement, so it is meaningless to be seen as an objective. Moreover, the indicators in HOM are important for the cavity of the 4th generation synchrotron radiation sources, in which the ${R/Q}$ should be minimized, and the frequency should be maximized. As a result, four indicators shown in Eq. (15) are selected as the objectives of the optimization. Note that the indicators with * should be minimized, so they are transferred to satisfy the mathematical model mentioned above. This is shown in Eq. 15 as well. Besides, HOM is the first higher order mode we meet during the calculation. The $\delta$ in equality constraint is 0.05.

14 $$R/Q = \frac{{R_{a} }}{Q}$$

15 $$\begin{gathered} \begin{array}{*{20}l} {{\text{min}}} & {\vec{F}\left( {\vec{x}} \right) = \left( {\begin{array}{*{20}l} {F_{1} \left( {R/Q_{{{\text{FM}}}} \left( {\vec{x}} \right)} \right),} \\ {F_{2} \left( {R_{{\text{a FM}}} \left( {\vec{x}} \right)} \right),} \\ {F_{3} \left( {f_{{{\text{HOM}}}} \left( {\vec{x}} \right) - f_{FM} \left( {\vec{x}} \right)} \right),} \\ {F_{4} \left( {{*}R/Q_{{{\text{HOM}}}} \left( {\vec{x}} \right)} \right)} \\ \end{array} } \right),} \\ \end{array} \hfill \\ \qquad {\text{s.t. }}f_{FM} - 499.65 = 0, \hfill \\ \qquad {\text{*A}} = \max \left( {\text{A}} \right) - {\text{A}}. \hfill \\ \end{gathered}$$

When the range of FM frequency in 1000 initial individuals is between 294.44 MHz and 954.05 MHz, which is 6600 times larger than the constraint, approximately 300 feasible individuals are found after evaluating 2000 individuals within 40 evaluated generations. This algorithm takes about 34 h totally when two CST processions work simultaneously.

In order to finish following discussion, a suitable *k* (which is mentioned in Eq. 8) is set as 0.1. Fit sizes of two groups of parents are set as well, which are 100 and 50 to feasible and infeasible groups respectively. The trends of changing *k* and the sizes of two groups of parents are discussed in Supplementary materials. These parameters in DNMOGA mainly influence the speed of convergence, while the performance of nondominated individuals would be affected tremendously only with harsh settings.

**Figure 4:**
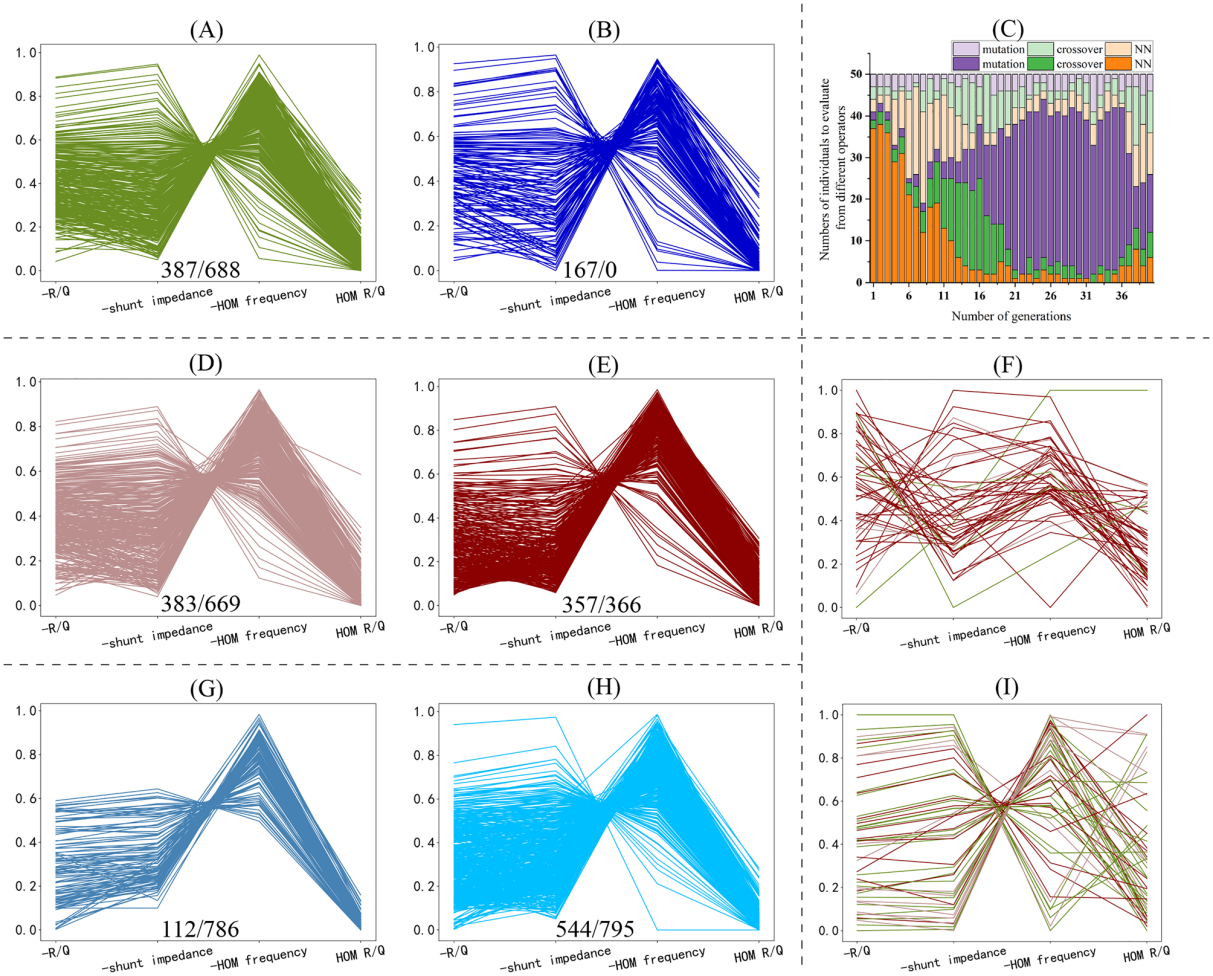
The results of DNMOGA. (**a**) Result of DNMOGA (k = 0.1, sizes of the two groups of parents = (100,50), initial size = 1000, generation = 40, $\vec{\text H}$  = (0,0,0,0), using high accurate NN). (**b**) Result of DNMOGA (k = 0.1, size of parents = 150, initial size = 1000, generation = 40, $\vec{\text H}$ = (0,0,0,0), using high accurate NN). (**c**) Redistribution quantities of evaluated individuals in Fig. 4a. The darker bars represent the numbers of individuals which are selected as parents of the next generation, and the lighter bars represent the numbers of individuals which are not selected. (**d**) Result of DNMOGA (k = 0.1, sizes of the two groups of parents = (100, 50), initial size = 1000, generation = 40, $\vec{\text H}$ = (5,1,1,0), using high accurate NN). (**e**) Result of DNMOGA (k = 0.1, sizes of the two groups of parents = (100,50), initial size = 1000, generation = 40, $\vec{\text H}$ = (10,1,1,0), using high accurate NN. (**f**) 50 ranked and selected individuals that come from Fig. 4a,d,e according to the R/Q of the FM. (**g**) Result of DNMOGA (k = 0.1, sizes of the two groups of parents = (100, 50), initial size = 1000, generation = 40, $\vec{\text H}$ = (0,0,0,0), using low accurate NN). (**h**) Result of DNMOGA (k = 0.1, sizes of the two groups of parents = (100,50), initial size = 1000, generation = 60, $\vec{\text H}$ = (0,0,0,0), using low accurate NN). (**i**) 50 ranked and selected individuals that come from Fig. 4a,d,e according to the R/Q of the HOM. Note that the Fig. 4a,b,d,e,g,h are the parallel coordinate plots of the feasible nondominated fronts in the last generation, in which every line represents an individual. The first objective in these subfigures is the R/Q of the FM, and the second is the Ra of the FM. The two values at the bottom of subfigures represent the numbers of feasible and infeasible nondominated individuals of the last generation respectively.

The results of experiments are shown in Fig. 4. The first comparison is about the two groups of parents and the general way of setting parents. In Fig. 4a,b, both the constants (mentioned above) of setting the total number of parents are the same as 150. The performance of nondominated individuals produced by the two groups of parents is approximately like that with the general method, but the former one creates a larger size of nondominated front.

Figure 4c illustrates the dynamic redistribution of evaluated individuals. As the algorithm goes, the penalty is stricter so that the individuals from NN performs worse in fitness values. As a result, less individuals from NN are picked up, and this is the reason why DNMOGA performs better than others when the accurate of NN is dissatisfied.

The results of DNMOGA with two different preferences are shown as well, and their $\overrightarrow{H}$ vectors (described in Eq. 12) are set as $(\mathrm{5,1},\mathrm{1,0})$ (Fig. 4d) and $(\mathrm{10,1},\mathrm{1,0})$ (Fig. 4e) respectively. The four values in $\overrightarrow{H}$ correspond with the four objectives ranging from F1 to F4 in turn. When the preference of F1 is improving gradually, more individuals that perform good in this objective are obtained. In Figs. 4f,i, individuals from Fig. 4a,d,e are mixed, and all these individuals are ranked by F1 and F4 respectively. From these figures, individuals generated with $(\mathrm{10,1},\mathrm{1,0})$ perform well in F1 and poorly in F4.

By comparing the results of DNMOGA with two different NN models, it is concluded that the accuracy of NN only influences the convergence speed. When the total number of evaluated generations is 40, the performance of DNMOGA with ANN is not good (Fig. 4g). But if continuing to evaluate 20 generations (Fig. 4h), it is nearly the same as the result of 40 total generations with a better NN model (Fig. 4a).

**Figure 5:**
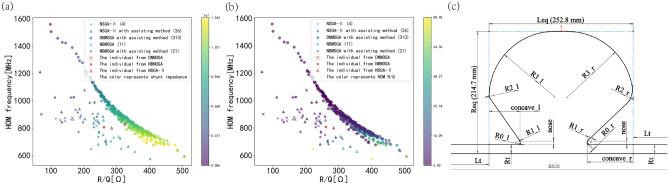
Comparing the results of different algorithms. (**a**) The scatter graphs of feasible and nondominated fronts from different algorithms. The colored z-axis represents the R_a FM_. The abscissa and ordinate represent R/Q of the FM and HOM frequency respectively, and the number in brackets represents how many feasible and nondominated individuals exist in each algorithm. The red shapes represent the individuals picked up from these algorithms. (**b**) The scatter graphs of feasible and nondominated fronts from different algorithms, in which the colored z-axis represents the R/Q_HOM_. (**c**) The geometric parameters of the SS cavity and the shape of an individual picked up from Fig. 4a.

The results of different algorithms (NSGA-II, DNMOGA and the NBMOGA in^15^) are shown in Fig. 5a,b, while the method proposed by^29^ is combined in NSGA-II and NBMOGA to deal with constraints. All these experiments evaluated 50 individuals per generation, and the total number of individuals is 3000. The initial population and the numbers of the total generations in NSGA-II are the same as DNMOGA, while these values are 50 and 500 for NBMOGA to keep the same number of estimated generations as DNMOGA (the training of NN in NBMOGA begins at the 10th generation). From the result of Fig. 5a,b, adjusting frequency is a useful assisting method to obtain more feasible individuals, while the gaps in the distributions and sizes of nondominated front between the two algorithms and DNMOGA is clear.

Then the individuals that have the similar frequency of the HOM are picked up from different algorithms, and their locations in the nondominated fronts are signaled on Fig. 5a,b. The indicators of these individuals are shown in Table 2, in which the advantage of DNMOGA can be discovered. The R/Q_FM_ is improved by about 24% and 14% compared with using the NBMOGA and NSGA-II, while the R_a FM_ is increased by approximately 55 and 22% respectively. Besides, only the R/Q _HOM_ that comes from the individual of DNOMGA tends to zero, which is beneficial for further analysis of HOM. The geometric parameters of the individual from DNMOGA are shown in Fig. 5c.

**Table 2:** The indicators of individuals picked up from the feasible and nondominated fronts of last generation.

Algorithm	R/Q_FM_ [Ω]	R_a FM_ [MΩ]	*f*_HOM_ [MHz]	R/Q_HOM_ [Ω]
DNMOGA	324.72	12.15	826.75	8.28E-7
NBMOGA	261.09	7.82	806.46	9.46
NSGA-II	284.54	9.93	800.56	18.58

Some benchmark optimization problems, such as CEC2009, DTLZ, and CMOP, are also used to validate the performance of DNMOGA, and the results have been added in the Supplementary materials.

## Discussion

Various machine learning models have potential to be combined into MOGAs to solve multi-objective optimization problems, but the way to combine is the key factor to influence the performance. In this paper, DNMOGA in which NN is dynamically used in a novel way of combination, is proposed and demonstrated. It’s good at dealing with the optimizing problem in physics, especially the complex questions with constraints and preference. It is easy to find the advantage of DNMOGA compared to NBMOGA and NSGA-II through the design of RF cavity. At the same time, using assisting methods is functional to make individuals more feasible in problems with strict constraints, while these methods are easier to operate in physically meaningful problems.

Not only can all kinds of RF cavity optimizations be well handled, such as multi-cell cavity^11^ and heavy ion cavities^41^, but also other accelerator optimization are principally suitable, like free electron laser^5,6^, nonlinear beam dynamics^4^ and so on. If we look at optimization designs in other physics fields, such as radio apparatus^42^ and structural components of materials^43^, more questions would be solved. In aerospace, MOGAs have been used to optimize 3D Wing-Shape, but response surface methodology is executed to meet the limited computing resources^44^; if suitable estimator is combined into MOGAs, the limit of calculation resource might be solved. All in all, it is obvious to see that combining machine learning and MOGAs have great potential to be dug out, the DNMOGA is one of the excellent methodology output.

## Methods

### Actual evaluator

The actual evaluator in these experiments is CST Studio Suite^45^, a software that can parametrically produce 3D models and obtain the electromagnetic field of the cavity by finite element analysis. The powerful post-processing functions in it can calculate various indicators. In this experiment, the mesh of the finite element analysis is set as 20 cells per wavelength.

### Calculation facility

The calculation facility used is a workstation with Xeon W-2265 CPU and 64 GB memory, and it takes about 45 s for this workstation to evaluate a cavity.

## Supplementary Information

Supplementary Information.

## Data Availability

The raw data generated during the current study is available in the github repository, https://github.com/PeilinWangWHU/Combining-multi-objective-genetic-algorithm-and-neural-network-dynamically.

## References

[1] Deb K, Pratap A, Agarwal S, Meyarivan T (2002). A fast and elitist multiobjective genetic algorithm: NSGA-II. IEEE Trans. Evol. Comput..

[2] Zhang Q, Li H (2007). MOEA/D: A multiobjective evolutionary algorithm based on decomposition. IEEE Trans. Evol. Comput..

[3] Zitzler E, Thiele L (1999). Multiobjective evolutionary algorithms: A comparative case study and the Strength Pareto approach. IEEE Trans. Evol. Comput..

[4] Gao W, Wang L, Li W, Beams.  (2011). Simultaneous optimization of beam emittance and dynamic aperture for electron storage ring using genetic algorithm. Phys. Rev. Spec. Top.-Accel..

[5] Yan J, Deng H (2019). Generation of large-bandwidth x-ray free electron laser with evolutionary many-objective optimization algorithm. Phys. Rev. Accel. Beams.

[6] Wu J (2017). Multi-dimensional optimization of a terawatt seeded tapered free electron laser with a multi-objective genetic algorithm. Nucl. Instrum. Methods Phys. Res. Sect. A-Accel. Spectrom. Detect. Assoc. Equip..

[7] Hofler A (2013). Innovative applications of genetic algorithms to problems in accelerator physics. Phys. Rev. Spec. Top.-Accel..

[8] Courant R, Hilbert D (1954). Methods of mathematical physics. Bull. Am. Math. Soc..

[9] Kranjčević M, Adelmann A, Arbenz P, Citterio A, Stingelin L (2019). Multi-objective shape optimization of radio frequency cavities using an evolutionary algorithm. Nucl. Instrum. Methods Phys. Res., Sect. A.

[10] Kranjčević M, Zadeh SG, Adelmann A, Arbenz P, Van Rienen U (2019). Constrained multiobjective shape optimization of superconducting rf cavities considering robustness against geometric perturbations. Phys. Rev. Accel. Beams.

[11] Luo T (2019). RF design of APEX2 two-cell continuous-wave normal conducting photoelectron gun cavity based on multi-objective genetic algorithm. Nucl. Instrum. Methods Phys. Res. Sect. A.

[12] Li Z-Q, Zhang C (2003). Study of heavily damped SC RF cavity. Chin. Phys. C.

[13] Zheng H-J, Gao J, Liu Z-C (2016). Cavity and HOM coupler design for CEPC. Chin. Phys. C.

[14] Kranjcevic M, Riemann B, Adelmann A, Streun A (2021). Multiobjective optimization of the dynamic aperture using surrogate models based on artificial neural networks. Phys. Rev. Accel. Beams.

[15] Wan J, Chu P, Jiao Y (2020). Neural network-based multiobjective optimization algorithm for nonlinear beam dynamics. Phys. Rev. Accel. Beams.

[16] Edelen A (2020). Machine learning for orders of magnitude speedup in multiobjective optimization of particle accelerator systems. Phys. Rev. Accel. Beams.

[17] Ben Said L, Bechikh S, Ghedira K (2010). The r-Dominance: A new dominance relation for interactive evolutionary multicriteria decision making. IEEE Trans. Evol. Comput..

[18] Molina J, Santana LV, Hernandez-Diaz AG, Coello CAC, Caballero R (2009). g-dominance: Reference point based dominance for multiobjective metaheuristics. Eur. J. Oper. Res..

[19] Marhauser, F., Weihreter, E., Dykes, D. & McIntosh, P. in *PACS2001. Proceedings of the 2001 Particle Accelerator Conference (Cat. No. 01CH37268).* 846–848 (IEEE).

[20] Lippmann R (1987). An introduction to computing with neural nets. IEEE ASSP Mag..

[21] Widrow B, Lehr MA (1990). 30 years of adaptive neural networks: Perceptron, madaline, and backpropagation. Proc. IEEE.

[22] Girosi F, Poggio T (1990). Networks and the best approximation property. Biol. Cybern..

[23] Rumelhart DE, Hinton GE, Williams RJ (1986). Learning representations by back-propagating errors. Nature.

[24] Khan S (2021). Transformers in vision: A survey. ACM Comput. Surv..

[25] Vaswani, A. et al. Attention is all you need. In *Advances in Neural Information Processing Systems*, Vol. 30 (eds. Guyon, I. *et al.*) (Curran Associates, Inc., 2017).

[26] Wang Y, Li JP, Xue XH, Wang BC (2020). Utilizing the correlation between constraints and objective function for constrained evolutionary optimization. IEEE Trans. Evol. Comput..

[27] Tessema B, Yen GG (2009). An adaptive penalty formulation for constrained evolutionary optimization. IEEE Trans. Syst. Man Cybern. Paart A Syst. Hum..

[28] Liu JJ, Teo KL, Wang XY, Wu CZ (2016). An exact penalty function-based differential search algorithm for constrained global optimization. Soft. Comput..

[29] Deb K (2000). An efficient constraint handling method for genetic algorithms. Comput. Meth. Appl. Mech. Eng..

[30] Runarsson TP, Yao X (2000). Stochastic ranking for constrained evolutionary optimization. IEEE Trans. Evol. Comput..

[31] Cai ZX, Wang Y (2006). A multiobjective optimization-based evolutionary algorithm for constrained optimization. IEEE Trans. Evol. Comput..

[32] Wang Y, Cai ZX, Guo GQ, Zhou YR (2007). Multiobjective optimization and hybrid evolutionary algorithm to solve constrained optimization problems. IEEE Trans. Syst. Man Cybern. Part B-Cybern..

[33] Jiao LC, Li L, Shang RH, Liu F, Stolkin R (2013). A novel selection evolutionary strategy for constrained optimization. Inf. Sci..

[34] Peng CD, Liu HL, Gu FQ (2018). A novel constraint-handling technique based on dynamic weights for constrained optimization problems. Soft. Comput..

[35] Wang Y, Cai Z, Zhou Y, Zeng W (2008). An adaptive tradeoff model for constrained evolutionary optimization. IEEE Trans. Evol. Comput..

[36] Fan Q, Yan X (2012). Differential evolution algorithm with co-evolution of control parameters and penalty factors for constrained optimization problems. Asia-Pac. J. Chem. Eng..

[37] Hou ZL, He C, Cheng R (2020). Reformulating preferences into constraints for evolutionary multi- and many-objective optimization. Inf. Sci..

[38] Hansen WG (1959). How accessibility shapes land use. J. Am. Inst. Plann..

[39] McKay MD, Beckman RJ, Conover WJ (2000). A comparison of three methods for selecting values of input variables in the analysis of output from a computer code. Technometrics.

[40] Feng H (2020). Proposed design and optimization of a higher harmonic cavity for ALS-U. Rev. Sci. Instrum..

[41] Yamada, S. in *Proc. 1981 Linac Conf.* (International Atomic Energy Agency).

[42] Lewis, A., Weis, G., Randall, M., Galehdar, A. & Thiel, D. in *2009 IEEE Congress on Evolutionary Computation.* 1486–1492 (IEEE).

[43] Todoroki, A. & Sekishiro, M. in *AIAA Infotech@ Aerospace 2007 Conference and Exhibit.* 2880 (Aerospace Research Central).

[44] Elham A, van Tooren MJ (2014). Weight indexing for wing-shape multi-objective optimization. AIAA J..

[45] Studios, C. M. & CST, M. CST Microwave studio. *CST Studio Suite* (2008).

