# Supplementary material for: Combining multi-objective genetic algorithm and neural network dynamically for the complex optimization problems in physics
Source: Sci Rep. 2023 Jan 17;13:880. doi: 10.1038/s41598-023-27478-7 (PMC10279691; doi:10.1038/s41598-023-27478-7)
Supplement: Supplementary file 1 — Supplementary Information. [file 41598_2023_27478_MOESM1_ESM.docx]

**Combining** **multi-objective** **genetic** **algorithm** **and** **neural** **network** **dynamically** **for** **the** **complex** **optimization** **problems** **in** **physics**

**Peilin Wang**1**,** **Kuangkuang Ye**1**,** **Xuerui Hao**1**,** **and** **Jike Wang**1, *

1 The Institute for Advanced Studies, Wuhan University, Wuhan 430072, China

* jike.wang@whu.edu.cn

**Design of the NN models**

In order to illustrate the relationship of the optimized performance and the accuracy of neural network (NN), two different models are used. One of them is a simple artificial neural network (ANN) which has 5 neurons within one hidden layer^1-4^, while the other one combining ANN and Transformer has better accuracy.

sss
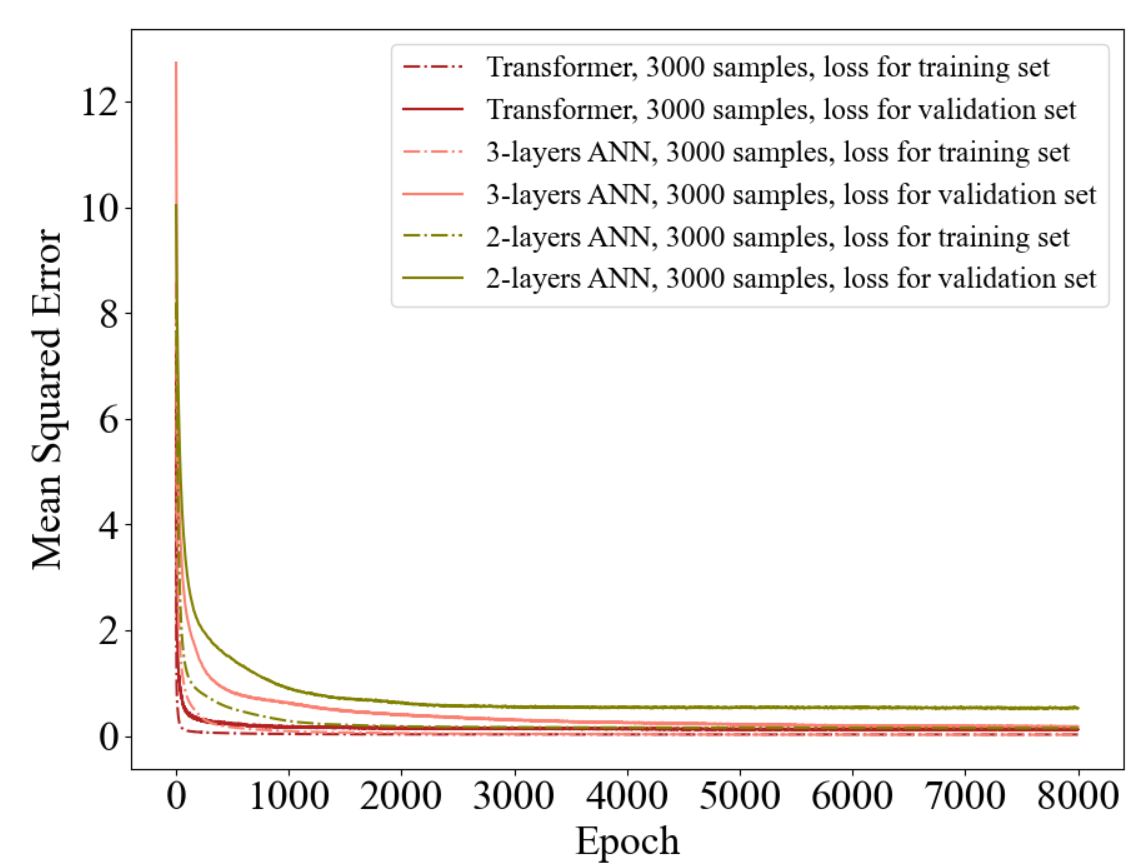


**Figure S1.** Comparison of the speed of convergence between the combination of the two models and the pure ANN. The Mean Squared Error is a loss function to illustrate the distance between estimated value and label, and the less loss means better performance of NN. Besides, the NN converges when the loss stops decreasing.

The overall idea of designing the model with better accuracy is to improve the relational inductive biases^5^. ANN could be an efficient solution to the regression problems, while the Transformer which has stood out in the natural language processing (NLP)^6^ in recent years has the outstanding ability of generalization^7^. Consequently, the Transformer is combined with ANN to improve the accuracy of estimation when the size of training set is small. In the specific operations, the first step is normalizing the decision variables, and then these variables are put into a vector list by executing positional encoding. Because of the symmetry of cavity, these decision variables exist symmetrically, and the paired variables are placed in the same vectors. As for the hyperparameters of the Transformer, the length of each vector is 2 because of the symmetry of decision variables, while the number of heads for multi-head attention that originally was 6 is set as 2 to reduce the number of training parameters. Then, the Transformer is combined with a 2-layer ANN to build the regression prediction model used in this study.

As for some details of these models, ELU activation is used in all neurons except the last layer of ANN which adopts the linear activation function. Besides, a quarter of the samples are used as the validation set to supervise the training process.

According to the **Table S1**, the Transformer combined with ANN has better performance. To further illustrate the advantage of it, another ANN model is used. This model has 3 layers, and the first two layers of it have 12 and 8 neurons respectively, which means this model has similar number of trainable parameters to the combined model. From **Figure S1** and **Table S1**, the combined model converges faster and
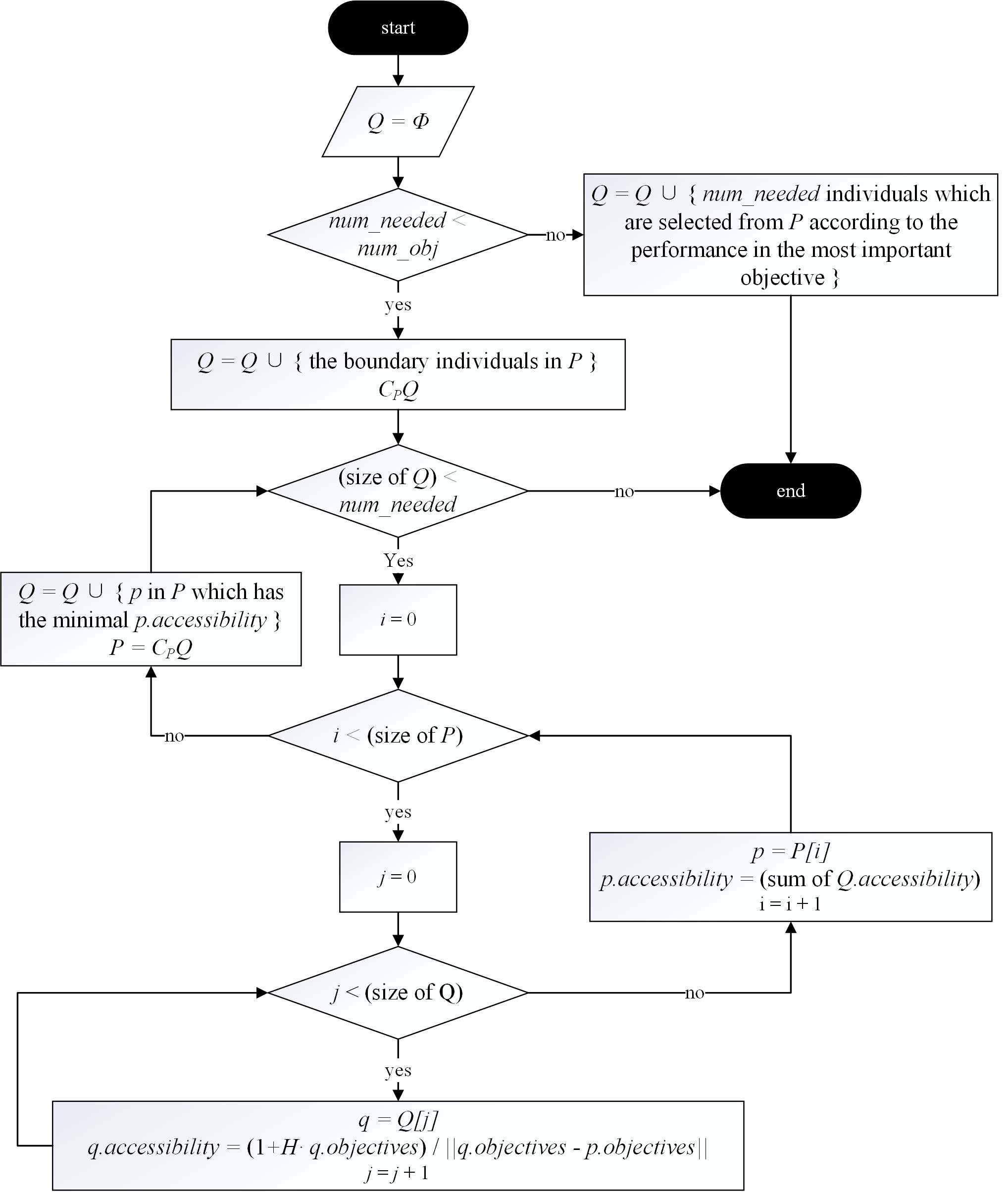
performs better in a small training set.

**Figure S2.** The flow chart of accessibility algorithm.

**Table S1.** The R^2^ in different models.

| Training sets and model | | Number of  trainable parameters | R^2^ | | | | |
| --- | --- | --- | --- | --- | --- | --- | --- |
|  |  |  | Resonant frequency | R/Q | Shunt impedance | Q factor | HOM frequency |
| 3000  samples | Transformer with ANN | 265 | 0.987 | 0.985 | 0.989 | 0.988 | 0.984 |
|  | 3-layers ANN | 281 | 0.970 | 0.983 | 0.987 | 0.991 | 0.975 |
|  | 2-layers ANN | 114 | 0.923 | 0.954 | 0.957 | 0.969 | 0.942 |

**Accessibility algorithm**

The fake code of accessibility algorithm is shown in **Table S2**, and the flow chart is shown in **Figure S2**.

**Table S2.** The fake code of the accessibility algorithm.

| ***Algorithm: accessibility algorithm (P, num_needed,*** $\vec{\text{H}\text{ }}$***)*** |
| --- |
| **Input: *P* (the nondominated front)** |
| ***num_needed* (number of parents needed)** |
| $\vec{\text{H}\text{ }}$ **(preference vector)** |
| **Output: *Q* (parents set of next generation)** |
| *1: Q =* $\emptyset$ |
| *2:* if *num_needed < num_obj*: ***//num_obj* represents the number of objectives** |
| *3: Q = Q* ∪ { *num_needed* individuals which are selected from *P* according to the performance in the most important objective |
| *4:*  else: |
| *5: Q = Q* ∪ { the boundary individuals in *P* } |
| *6: P =* Ⅽ*_P_Q* ***//*delete the boundary individuals from *P*** |
| *7:* while (size of *Q*) *< num_needed*: |
| *8:*  for *p* in *P*: ***//p* is individual in *P*** |
| *9:*  for *q* in *Q*: |
| *10: q.accessibility =* (1*+*$\vec{\text{H}\text{ }}\text{∙}\text{ }\vec{\text{q.objectives}}$) / *\|\|*$\vec{\text{q.objectives}}$ *-* $\vec{\text{p}\text{.objectives}}$*\|\|* |
| *11: p.accessibility =* sum of *Q.accessibility* |
| *12: Q = Q* ∪ { *p* in *P* which has the minimal *p.accessibility* } |
| *13: P =* Ⅽ*_P_Q* |
| *14:* return*Q* |

**The complete process of DNMOGA**

The fake code of the complete process is shown in **Table S3**.

**Table S3.** The fake code of the complete process of DNMOGA.

| ***Algorithm: the complete process of DNMOGA ()*** |
| --- |
| **Output: *parents*** **//excellent individuals in last generation** |
| *1: dec_val =* execute LHS () **// Latin hypercube sampling** |
| *2: all_ind =* simulate (*dec_val*) |
| *4:* train the NN model (*all_ind*) |
| *5: ind_sort =* execute fast non-dominated sort algorithm in two groups of parents (*all_ind*) |
| *6: parents =* execute accessibility algorithm in two groups of parents (*ind_sort*) |
| *7:*  while judge_end (): |
| *8: parents_for_pre = parents* |
| *9:* for *i* in 4: |
| *10: dec_val_for_pre =* execute crossover, mutation and LHS (*parents_for_pre*) |
| *11: ind_pre =* estimate with NN (*dec_val_for_pre*) |
| *12: ind_sort =* execute fast non-dominated sort algorithm (*ind_pre*) |
| *13: parents_for_pre =* pick up all the individuals in the nondominated front (*ind_sort*) |
| *14: dec_val_for_pre =* execute crossover, mutation and LHS (*parents_for_pre*) |
| *15: ind_pre =* predict with NN (*dec_val_for_pre*) |
| *16: ind_sort =* execute fast non-dominated sort algorithm (*ind_pre*) |
| *17: ind_1 =* execute accessibility algorithm (*ind_sort*) |
| *18: ind_2 =* execute crossover and mutation (*parents*) |
| *19: ind_simulated =* evaluate (*ind_1* ∪ *ind_2*) |
| *20: all_ind = all_ind* ∪ *ind_simulated* |
| *21: ind_sort =* execute fast non-dominated sort algorithm in two groups of parents (*all_ind*) |
| *22: parents =* execute accessibility algorithm in two parent groups (*ind_sort*) |
| *23:* train the NN model (*all_ind*) |
| *24:*  dynamically redistribute the numbers of individuals for the next generation (*parents* and *the redistributed numbers used in this generation*) |
| *25:* return *parents* |

**Nonlinear transformations between geometry parameters and independent variables**

There are totally 15 geometric parameters in the SS cavity (cf. **Figure S3 (a and b)**), in which the *Rt*, *Lt* are not what we care about and consequently fixed. Instead of the 13 geometry parameters that we concerned, 13 independent variables are set as decision variables in DNMOGA, and these variables can be set as random values by LHS in the given space regardless of geometry limits. These independent variables are named *ran1*, *ran2*, *ran3_r*, *ran3_l*, *ran4*, *ran5_r*, *ran5_l*, *ran6_r*, *ran6_l*, *ran7_r*, *ran7_l*, *ran8_r*, *ran8_l*, respectively, while their ranges are shown in **Table S4**. The process of transformation from the independent variables to geometry parameters is shown below.

**Table S4.** The ranges of decision values.

| Variable | ran1 | ran2 | ran3_r | ran3_l | ran4 | ran5_r | ran5_l |
| --- | --- | --- | --- | --- | --- | --- | --- |
| Lower bound | 100 | 0.01 | 0.05 | 0.05 | 1 | 0.15 | 0.15 |
| Upper bound | 200 | 0.95 | 0.95 | 0.95 | 250 | 0.95 | 0.95 |
| Variable | ran6_r | ran6_l | ran7_r | ran7_l | ran8_r | ran8_l |  |
| Lower bound | 0.05 | 0.05 | 0.05 | 0.05 | 0.05 | 0.05 |  |
| Upper bound | 0.95 | 0.95 | 0.95 | 0.95 | 0.70 | 0.70 |  |

We first define *Req* as the sum of an independent variable and *Rt*. Besides, the *nose* is smaller than the difference of *Req* minus *Lt*:

|  | $R\text{eq}=ran1+Rt$, | (S1) |
| --- | --- | --- |
|  | $nose=ran2(Req-Rt)$. | (S2) |

*R3_l*, *R3_r* should be smaller than the difference of *Req* minus the sum of *Rt* and nose:

|  | $R{3\_l}=\left( 1-ran{3\_l} \right)\left( Req-Rt-nose \right),$ | (S3) |
| --- | --- | --- |
|  | $R{3\_r}=\left( 1-ran{3\_r} \right)\left( Req-Rt-nose \right).$ | (S4) |

The *Leq* equals to the sum of an independent variable plus twice the maximum value of *R3*:

|  | $Leq=2\max(R3\_l,R3\_r)+ran4$. | (S5) |
| --- | --- | --- |

The *concave_l* and *concave_r* are smaller than half of *Leq*:

|  | $concave\_l=ran5\_l\cdot Leq/2$, | (S6) |
| --- | --- | --- |
|  | $concave\_r=ran5\_r\cdot Leq/2$. | (S7) |

Calculating the maximum values of *R2_r* and *R2_l* is very complex, while the relative positions for both *R2_r* and *R2_l* to the surrounding geometric parameters are the same. Consequently, *R2_r* and *R2_l* can be replaced by *R2* to be further described. We assume the largest *R2* at one side exists when the arc of this *R2* is tangent to the top of the cavity, as the red line shown in the **Figure S3c**. In this scenario, the maximum value of *R2* can be expressed by following equations:

|  | $\cos\alpha=\frac{R3-temp\_x}{temp\_a} ,$ | (S8) |
| --- | --- | --- |
|  | $\sin\alpha=\frac{temp\_b}{R3-temp\_a\cdot\sin\alpha} ,$ | (S9) |
|  | $temp\_x= temp\_a+temp\_b,$ | (S10) |

in which $temp\_x$ is the largest *R2*. The $\alpha$ can be expressed as

|  | $\alpha=\arctan\left( \frac{ran3\left( Req-Rt-nose \right)}{concave} \right) .$ | (S11) |
| --- | --- | --- |

As a result, the *R2_l* and *R2_r* can be calculated as:

|  | $R{2\_l}=ran{8\_l}\cdot R{3\_l}\frac{\sin(\alpha\_l)+\cos(\alpha\_l)}{1+\cos(\alpha\_l)},$ | (S12) |
| --- | --- | --- |
|  | $R{2\_r}=ran{8\_r}\cdot R{3\_r}\frac{\sin(\alpha\_r)+\cos(\alpha\_r)}{1+\cos(\alpha\_r)}.$ | (S13) |

Because of the complex calculation as well, we assume the maximum values of both *R1_r* and *R1_l* exist when they are vertical to the tube (cf. the purple line in **Figure S3c**). By calculating following equations:

|  | $\frac{nose}{temp\_d}=\frac{R1\_max}{R1\_max+temp\_c} ,$ | (S14) |
| --- | --- | --- |
|  | $\sin\alpha=\frac{nose}{temp\_d} ,$ | (S15) |
|  | $\tan\alpha=\frac{nose}{temp\_c} ,$ | (S16) |

the *R1_r* and *R1_l* can be calculated as

|  | $R{1\_l}=ran{7\_l}\cdot nose\frac{cos(\alpha\_l)}{1-\sin(\alpha\_l)},$ | (S17) |
| --- | --- | --- |
|  | $R{1\_r}=ran{7\_r}\cdot nose\frac{cos(\alpha\_r)}{1-\sin(\alpha\_r)}.$ | (S18) |

Finally, *R0_l* and *R0_r* should be smaller than the distance between tube and the center of the arc of *R1*:

|  | $R0\_l=ran6\_l(1-ran{7\_l})nose,$ | (S19) |
| --- | --- | --- |
|  | $R0\_r=ran6\_r(1-ran{7\_r})nose.$ | (S20) |


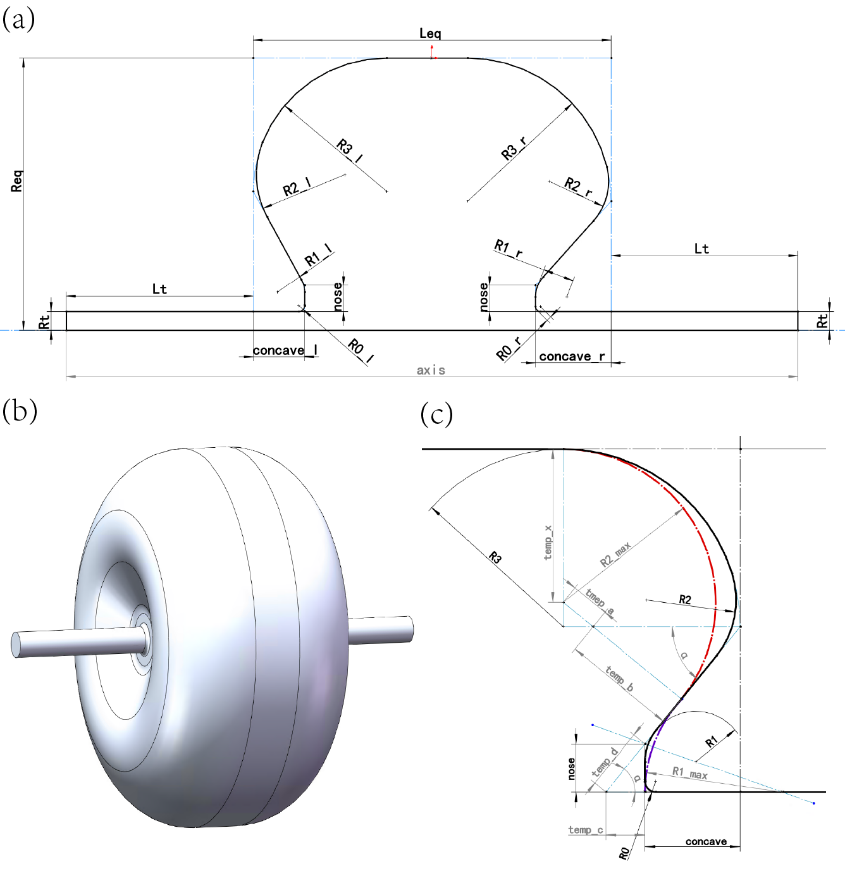


**Figure S3.** **(a)** The shape and geometry parameters of SS cavity. The black lines are the cross-sectional view of the cavity structure, and the blue lines are the construction lines (these lines are used to aid in drawing). The black parameters in this subfigure are the driving size, while the gray parameters are driven size. **(b)** A 3D shape of SS cavity. **(c)** The assumed maxima of R1 and R2, and their relative positions to the surrounding geometric parameters. The positions for both sides of R1 (for both R1_r and R1_l) and R2 (for both R2_r and R2_l) can be expressed in this subfigure, and the purple and red lines are the largest R1 and R2 respectively.

**The analysis of performance when the *k* and sizes of two groups of parents change**

In the following experiments, some operations are the same to ensure that the results are comparable. The first one is $\vec{\text{H }}$, and it is set as (0,0,0,0). Secondly, the initial populations are all set as 1000. Following that, the NN models used in these experiments are the Transformer combined with ANN.

**Figure S4 (a to i)** show the different performance when the *k* changes, while the sizes of two groups of parents for feasible and infeasible populations are fixed as 100 and 50 in turn. When *k* tunes from 0.4 to 1.6 (cf. **Figure S4 (a to e)**), the speed for penalty to be serious increases gradually, and this decreases the number of competitive individuals during the optimization. As a result, the competitiveness of parents declines, which consequently leads to the worse border and size of the feasible nondominated front. The trend is different when the *k* is between 0.01 and 0.1 (cf. **Figure. S3 (f to i)**). In this range, little improvement in the performance of border of the feasible nondominated front can be seen when *k* drops, because there are always competitive individuals throughout the whole period of optimization when the penalty operation changes. In other words, this feature means that premature phenomenon hardly exists, and the results are closer to the global optima. Then it is worthy to discuss the sizes of the feasible and infeasible nondominated fronts when the *k* is in the range of 0.01 and 0.1. Although the size of infeasible front grows as the *k* decreases, there are less feasible individuals in the front, which means the penalty is too small and many individuals in the infeasible front are far away from the range of constraint.


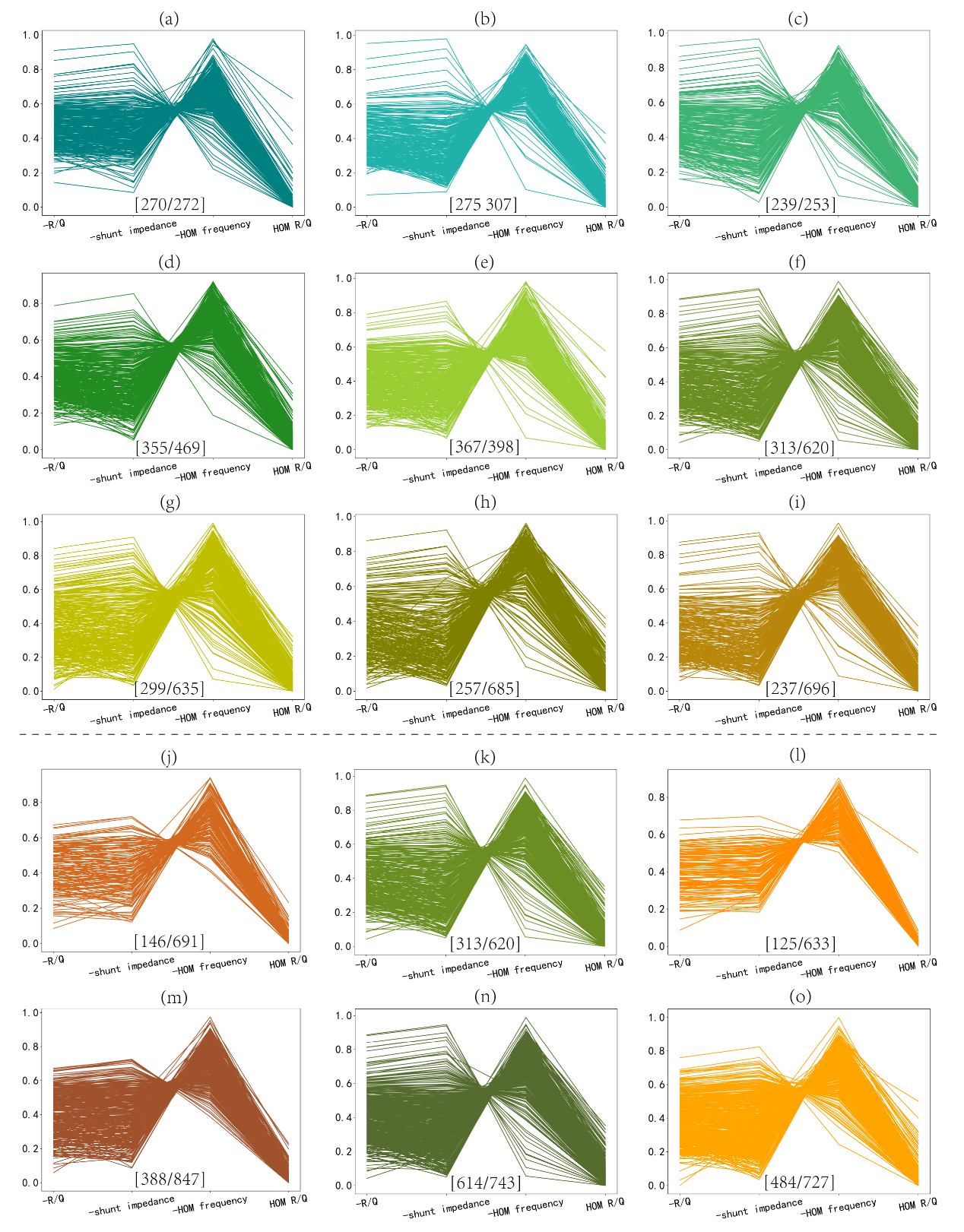


**Figure S4. The parallel coordinate plots of the feasible nondominated fronts.** [A/B] in these graphs represents that there are A feasible individuals and B infeasible individuals in the last nondominated front. **(a)**. Result of DNMOGA (k = 1.6, sizes of two groups of parents = (100,50) generation = 40). **(b)**. DNMOGA (k = 1.3, sizes of two groups of parents = (100,50), generation = 40). **(c)**. DNMOGA (k = 1, sizes of two groups of parents = (100,50), generation = 40). **(d)**. DNMOGA (k = 0.7, sizes of two groups of parents = (100,50), generation = 40). **(e)**. DNMOGA (k = 0.4, sizes of two groups of parents = (100,50), generation = 40). **(f)**. DNMOGA (k = 0.1, sizes of two groups of parents = (100,50), generation = 40). **(g)**. DNMOGA (k = 0.07, sizes of two groups of parents = (100,50), generation = 40). **(h)**. DNMOGA (k = 0.04, sizes of two groups of parents = (100,50), generation = 40). **(i)**. DNMOGA (k = 0.01, sizes of two groups of parents = (100,50), generation = 40). **(j)**. DNMOGA (k = 0.1, sizes of two groups of parents = (50,25), generation = 40). **(k)**. DNMOGA (k = 0.1, sizes of two groups of parents = (100,50), generation = 40). **(l)**. DNMOGA (k = 0.1, sizes of two groups of parents = (200,100), generation = 40). **(m)**. DNMOGA (k = 0.1, sizes of two groups of parents = (50,25), generation = 60). **(n)**. DNMOGA (k = 0.1, sizes of two groups of parents = (100,50), generation = 60). **(o)**. DNMOGA (k = 0.1, sizes of two groups of parents = (200,100), generation = 60).

Then the *k* is fixed as 0.1 to explore the influence of tuning the size of two groups of parents. The disadvantage of increasing the sizes of parents is that the number of generations required to make DNMOGA converge raises (cf. **Figure S4 (l and o)**), but the advantage of this increase can be discovered by comparing the fronts shown in **Figure S4 (m, n and o)**. Comparing with the front of **Figure S4m** whose sizes of parents are the smallest, the borders in the fronts produced by larger sizes of parents are better. Another phenomenon is that the borders in **Figure S4 (n and o)** are similar, which means these borders are close to the real border and could be hard for them to be better.

**Results of benchmark tests**

Some classic benchmark problems are utilized to further validate DNMOGA’s performance in several key aspects. DTLZ^8^ is usually used to test algorithm’s ability facing various problems without constraints. For example, DTLZ1 has too many local optima, while DTLZ2 is always used to test the uniformity of solutions in front. Different from DTLZ, CEC2009^9^ and LIR-CMOP^10^ have some problems involving constraints, among which LIR-CMOP8 has three constraints, while CEC2009CP1 and CEC2009CP2 whose difficulty of achieving convergence is different have the same number of constraints, and both are one. Here, DTLZ1, DTLZ2, CEC2009CP1, CEC2009CP2, and LIR-CMOP8 are tested, and the shapes of nondominated fronts in these problems as well as related parameters are respectively shown the figure and table below.

In this table, *m* and *l* are adjustable parameters which have been mentioned in Eq. 2, and the values of them are recommended by several papers^8-10^. Besides, *k* is a parameter described in Eq. 8. The values of *k* in CEC2009CP1 and CEC2009CP2 are set as the same as the one used in manuscript. However, this value for LIR-CMOP8 is decreased to 0.001 because of the special shape of feasible region in this problem. If *k* is set generally in this problem, algorithm will trend to converge early. From this figure, the solutions for DTLZ1, DTLZ2, as well as LIR-CMOP8 converge to their true Pareto front, but the results of CEC2009CP1 and CEC2009CP2 are not so good because the numbers of decision variables (*l*) are much more than the problems above.


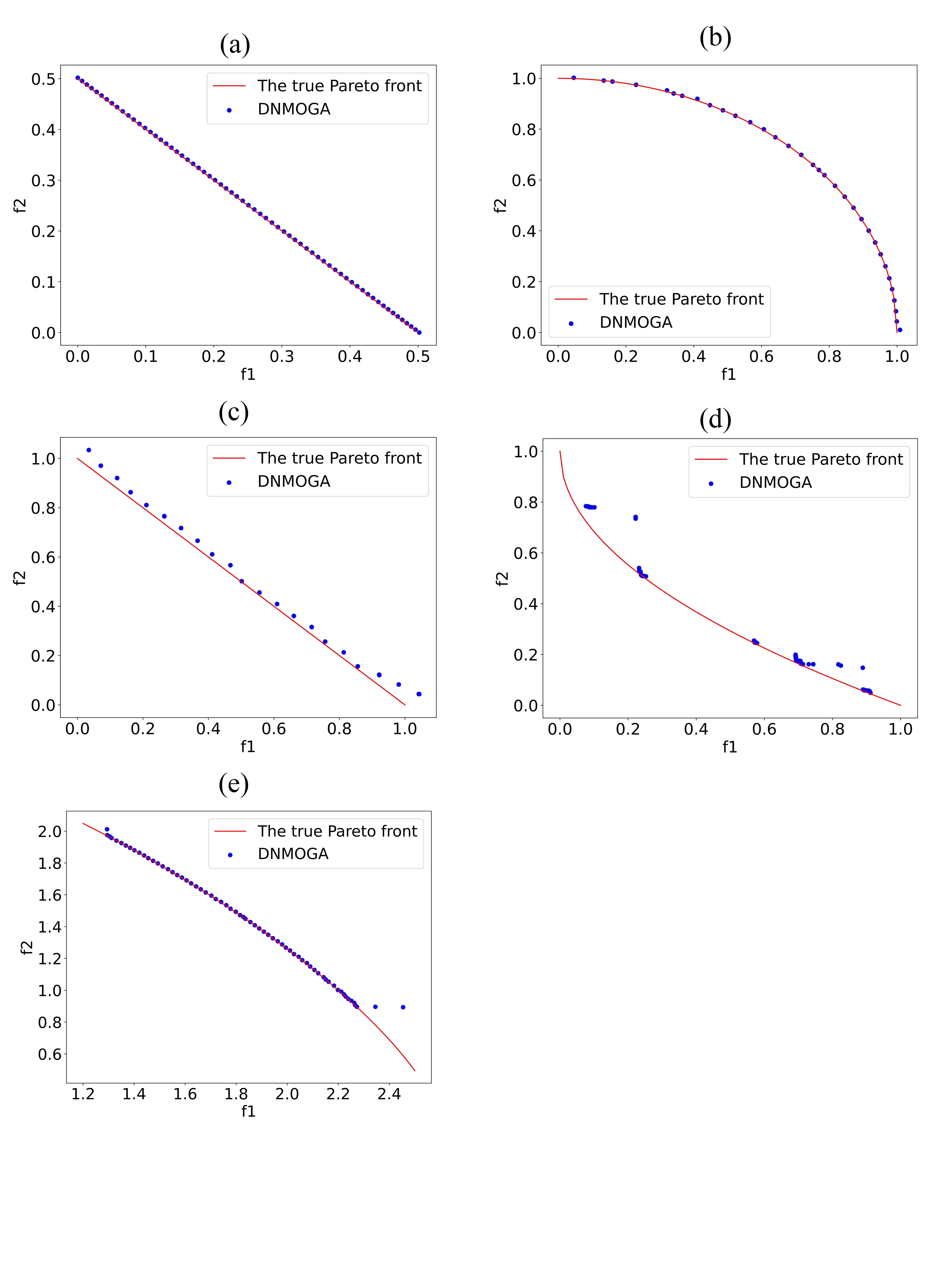


**Figure S5. The results of benchmark tests.**

**Table S5. The parameters of benchmark tests.**

| Problems | *m* | *l* | *k* | Number of initial individuals | Number of individuals per generation | Number of evaluated generations |
| --- | --- | --- | --- | --- | --- | --- |
| DTLZ1 | 2 | 4 | - | 10000 | 300 | 40 |
| DTLZ4 | 2 | 10 | - | 10000 | 300 | 100 |
| CEC2009CP1 | 2 | 30 | 0.1 | 10000 | 500 | 40 |
| CEC2009CP2 | 2 | 30 | 0.1 | 10000 | 500 | 40 |
| LIR-CMOP8 | 2 | 10 | 0.001 | 10000 | 300 | 70 |

**Reference**

1 Lippmann, R. An introduction to computing with neural nets. *IEEE Assp magazine* **4**, 4-22, doi:<https://doi.org/10.1109/MASSP.1987.1165576> (1987).

2 Widrow, B. & Lehr, M. A. 30 years of adaptive neural networks: perceptron, madaline, and backpropagation. *Proceedings of the IEEE* **78**, 1415-1442, doi:<https://doi.org/10.1109/5.58323> (1990).

3 Girosi, F. & Poggio, T. Networks and the best approximation property. *Biological cybernetics* **63**, 169-176, doi:<https://doi.org/10.1007/BF00195855> (1990).

4 Rumelhart, D. E., Hinton, G. E. & Williams, R. J. Learning representations by back-propagating errors. *nature* **323**, 533-536, doi:<https://doi.org/10.1038/323533a0> (1986).

5 Battaglia, P. W. *et al.* Relational inductive biases, deep learning, and graph networks. *arXiv:1806.01261*, doi:<https://doi.org/10.48550/arXiv.1806.01261> (2018).

6 Vaswani, A. *et al.* Attention is all you need. *Advances in neural information processing systems* **30** (2017).

7 Khan, S. *et al.* Transformers in vision: A survey. *ACM Computing Surveys*, doi:<https://doi.org/10.1145/3505244> (2021).

8 Deb, K., Thiele, L., Laumanns, M. & Zitzler, E. in *Evolutionary multiobjective optimization* 105-145 (Springer, 2005).

9 Zhang, Q. *et al.* Multiobjective optimization test instances for the CEC 2009 special session and competition. **264**, 1-30 (2008).

10 Fan, Z. *et al.* An improved epsilon constraint-handling method in MOEA/D for CMOPs with large infeasible regions. **23**, 12491-12510, doi:<https://doi.org/10.1007/s00500-019-03794-x> (2019).
